# Significant Reductions in Mortality in Hospitalized Patients with Systemic Lupus Erythematosus in Washington State from 2003 to 2011

**DOI:** 10.1371/journal.pone.0128920

**Published:** 2015-06-18

**Authors:** Louisa B Goss, Justin R Ortiz, Daryl M Okamura, Kristen Hayward, Christopher H Goss

**Affiliations:** 1 Department of Medicine, University of Washington, Seattle, Washington, United States of America; 2 Department of Global Health, University of Washington, Seattle, Washington, United States of America; 3 Department of Pediatrics, Division of Nephrology, University of Washington, Seattle, Washington, United States of America; 4 Seattle Children’s Research Institute, Seattle,Washington, United States of America; 5 Department of Pediatrics, Division of Rheumatology, University of Washington, Seattle, Washington, United States of America; 6 Department of Pediatrics, Division of Pulmonary, University of Washington, Seattle, Washington, United States of America; Baylor College of Medicine, UNITED STATES

## Abstract

**Background:**

Systemic lupus erythematosus (SLE or lupus) is an autoimmune multisystem disease. While a complete understanding of lupus’ origins, mechanisms, and progression is not yet available, a number of studies have demonstrated correlations between disease prevalence and severity, gender, and race. There have been few population based studies in the United States

**Objectives:**

To assess temporal changes in demographics and hospital mortality of patients with lupus in Washington State from 2003 to 2011

**Study Design:**

This study used data from the Healthcare Cost and Utilization Project (HCUP), a patient information database, and data from the Washington State census to study a group of patients in the state. Lupus hospitalizations were defined as any hospitalization with an ICD-9-CM diagnosis code for systemic lupus erythematosus. Regression analysis was used to assess the effect of calendar time on demographics and hospital outcomes.

**Results:**

There were a total of 18,905 patients in this study with a diagnostic code for lupus. The mean age of the group was 51.5 years (95% CI: 50.6-52.3) in 2003 and 51.3 years (95% CI: 50.6-52.0) in 2011. The population was 88.6% female. Blacks were 2.8 times more likely to have a lupus hospitalization than whites when compared to the Washington population. While hospital mortality decreased during this eight year period (3.12% in 2003 to 1.28% in 2011, p=0.001) hospital length of stay remained statistically unchanged at an average of 4.9 days during that eight year period. We found a significant decrease in annual hospital mortality over the study period [odds ratio(OR): 0.92 per year, 95% CI 0.88-0.96, P<0.001]. Hospital mortality was higher in males (2.6% male death to 1.8% female death)

**Conclusions:**

In this large group of hospitalized lupus patients in Washington, hospital length of stay remained relatively stable over time but hospital mortality decreased by over 50% over the eight year study period.

## Introduction

Systemic lupus erythematosus (SLE or lupus) is a complex autoimmune disease due primarily to inappropriate innate and adaptive immune response resulting in global loss of self-tolerance with activation of autoreactive T- and B-cells. Despite its relative prevalence (roughly 1/100,000 people in the US) the etiology of the disease is still poorly understood.[1,2] The autoimmunity of lupus is thought to be triggered by a variety of factors including susceptibility of approximately 30 genetic loci, environmental changes, and other random events.[2] These factors can also trigger other variants of lupus including cutaneous lupus which only affects the skin and drug-induced lupus which can be cured by withdrawal of the offending agent. Lupus patients develop a variety of signs and symptoms including fever, fatigue, lymphadenopathy, alopecia, Raynaud’s phenomenon, photosensitivity, rashes (i.e. malar, discoid, livedo reticularis), oral and nasal ulcers, inflammatory arthritis, serositis (i.e. pleuritis, and pericarditis), renal involvement (nephritis, nephritic syndrome), strokes, seizures, cytopenias, and autoantibodies.[3] There are many medications that improve lupus (antimalarials, immunosuppressives, steroids, NSAIDs, some biologics), but there is no cure.[4] In lupus nephritis, there can be poor renal outcomes in 20% (requiring augmented immunotherapy, renal replacement therapy, or renal transplant).[5] Understanding the evolution of lupus requiring hospitalization can aid in determining if the disease severity and hospitalized mortality is changing over time.

For patients with lupus, genetics, gender, race, and environmental factors appear to play key roles.[1] In a nationwide survey, a higher prevalence of lupus nephritis was found among persons with lower socioeconomic status.[1] Other studies have noted an increased risk for end-stage renal disease and death among black or Hispanic lupus patients.[6] Studies of pediatric lupus have shown stable hospital length of stay, but decreasing hospital mortality.[7] Few studies have examined only lupus patients with severe outcomes or have used large administrative databases with known population denominators.[1,6,7] This study examines data from hospitalized lupus patients in Washington State and provides an update to earlier studies and may improve our understanding of how demographics and clinical outcomes of patient hospitalized with lupus are changing over time.

Our objective was to study the temporal changes in both demographics and mortality of hospitalized lupus patients from 2003 to 2011 in Washington State. We hypothesized that we would see no significant changes in demographics in these patients over this time period. We also hypothesized that given changes in medical management and medication regimens, the hospital mortality would fall.

## Methods

We had formal approval to use state level Healthcare Cost and Utilization Project (HCUP) data. The study examined hospital stays of lupus patients using 2003 to 2011 discharge data from the Washington State Inpatient Databases (SID), Healthcare Cost and Utilization Project (HCUP), Agency for Healthcare Research and Quality. Data are available from the Healthcare Cost and Utilization Project (HCUP) (the State Inpatient Database (SID) from Washington State) for researchers who meet the criteria for access to confidential data. This project was reviewed by the Institutional Review Board at the University of Washington Human Subjects Division and was granted formal human subjects exemption.

### Study Population

This study evaluates all patients hospitalized with lupus (defined as severe lupus) in Washington State from 2003 to 2011. We used patient information from the HCUP State Inpatient Database (SID) for Washington State from 2003 through 2011.[8] Subjects were identified using International Classification of Diseases, 9^th^ revision (ICD-9-CM) codes with either a primary or secondary diagnosis of lupus ICD-9-CM (710.0).[9] Others have also used this billing code to classify patients with lupus for epidemiologic research.[10]

### Variables

The SID has hospitalization level data with limited demographic variables: age at admission, calendar year, deaths, gender, and race. Race was categorized in the SID as white, black, Hispanic, Asian and Pacific Islander, or Native American. The race of each patient was based on self-report and was only available from 2008 through 2011. We studied hospital length of stay and death during hospitalization.

To augment data available in the database and establish the racial composition of Washington, we used data from the Washington State census during this time period. These values included the total annual population estimate of Washington during the study period and the racial composition of the state. These values were used to establish the effect of race on lupus hospitalizations. Because the SID data and census data categorize race/ethnicity differently, we had to adapt these variables to allow comparisons. While the SID database categorized patients into white, black, Hispanic, Asian or Pacific Islander, Native American, or other, the census categorized the population into white, African Americans, Asian, American Indian and Alaska native, Native Hawaiian and Pacific Islander, or two or more races. In addition, the census categorizes each individual as being non-white Hispanic or non-Hispanic regardless of race categorization. To compare racial distributions in the SID and census, we combined white and Hispanic values in the SID in order to make a meaningful comparison with the census data on whites. We deemed that the Asian or Pacific Islander category in the SID was comparable to the Asian and Native Hawaiian and Pacific Islander category in the census. We compared the Native American category in the SID to the American Indian and Alaska Native categories in the census. The “other race” category in SID was set to be equal to the two or more races category in the census. We did a separate comparison for ethnicity. We compared Hispanics in the SID to non-white Hispanics in the census. For a non-Hispanic comparison, we compared all the other races from the database to the non-Hispanic values from the Washington State census.

To address concerns of the specificity of a lupus diagnosis, we repeated our primary analyses restricting the data to just those patients with a primary diagnosis code for lupus. In addition to this sensitivity analysis, we also assessed the annual percentage of patients undergoing hemodialysis and mechanical ventilation. Hemodialysis was defined by the use of two ICD-9 procedure codes: 39.95 and 38.95. The use of mechanical ventilation was identified by the presence of two ICD-9 procedure codes: 96.71 and 96.72.[11] These procedure codes when formally assessed against the medical records in the US identified major or invasive procedures reasonably well. These variables were used to adjust for severity of illness. To further explore the role of severity of illness, we employed the Quan modification of the Deyo-Charlson comorbidity index to assess changing severity of illness over time.[12,13] The index from the Quan modification yields values from 0 to 33, with increasing scores reflecting increasing burden of comorbidities. The C-Statistic for the Deyo-Charlson Index is reported to be 0.842 for the ICD-9 based index compared to 0.878 for the ICD-9 Quan enhanced Deyo-Charlson Comorbidity Index.

### Statistics

We performed the primary analyses based on an *a priori* statistical analysis plan. Descriptive analyses were used to assess patient characteristics including distribution plots, means for normally distributed data, medians, and interquartile ranges. Formal missing data assessment was done for all of the key variables. Chi square statistic was used to compare categorical variables, Student’s t-test compared means between two groups, one sample test of proportions was used to establish the proportion of one outcome within a population, and Wilcoxon Rank Sum statistics to compare data that were not normally distributed. We used the Kruskal-Wallis rank test to assess differences in more than two groups of non-normally distributed variables with the addition of a non-parametric test of trend to assess changes across time. We also used logistic regression to assess changes in proportions over time, with calendar time as the independent variable and dichotomous outcome measures as the dependent variable. The data were de-identified so unique individuals could be represented in more than one hospitalization. Our unit of analysis was the hospitalization and we made the assumption that each hospitalization was independent. Statistically significant findings of these tests were defined by a p value ≤ 0.05. All analyses were performed using STATA version 11 (StataCorp LP, College Station, Texas).[14]

## Results

### Demographics

From 2003 through 2011, there were 18,905 hospitalizations with diagnostic codes for lupus in Washington, ranging from 1,743 hospitalizations in 2004 to 2,504 hospitalizations in 2011. During the study period, lupus hospitalization distribution by gender was unchanged at an approximate 9:1 ratio of females to males. We found no statistically significant changes in the gender ratio for the full time period (p = 0.79) (Table 1). The average age of the group in 2003 was 51.5 years (SD 0.42) and in 2011 it was 51.3 years (SD 0.37); we found no significant change in the age of the group over the study period (p = 0.5). The 9:1 ratio of females to males remained consistent despite the varying ages at which patients were admitted to the hospital (S1 Fig). However, males represented a higher proportion of the pediatric patients, particularly those under 10 years of age (S1 and S2 Figs). The average age of males was 50.2 years while the average age of females was 51.3 (p = 0.005).

**Table 1:** The percentage of lupus patients hospitalized every year by gender, age category, and hospital mortality and mean length of stay in Washington State from years 2003 through 2011.

	Calendar Year									
	2003	2004	2005	2006	2007	2008	2009	2010	2011	Average
Gender^¶^										
%male	10.8	11.5	11.3	10.7	11.5	11.5	11.9	11.2	12.4	11.5
% female	89.2	88.5	88.8	89.3	88.5	88.5	88.1	88.8	87.6	88.6
Age Category^‡^										
% ≤18 years	2.6	2.0	3.4	4.7	2.6	3.1	4.5	3.2	3.0	3.2
%> 18 years	97.4	98.0	96.6	95.3	97.4	96.9	95.5	96.8	97.0	96.8
% died during hospitalization *,^†^	3.1	2.0	2.3	2.3	1.8	1.4	1.7	1.8	1.3	1.9
Mean length of stay (days)^β^	5.1	4.9	4.9	4.9	4.9	5.0	5.2	4.6	5.0	4.9
% Requiring mechanical ventilation ^α^	3.4	2.8	3.0	2.9	2.4	2.8	3.6	3.1	2.8	3.0
% Requiring hemodialysis ^π^	7.0	5.8	7.4	6.1	5.9	6.5	5.2	6.4	6.0	6.2
Mean Quan Comorbidity Index ^Ω^	2.17	2.22	2.14	2.24	2.25	2.26	2.45	2.47	2.49	2.31

^¶^ OR of proportion of males being hospitalized did not change significantly over time (p = 0.14).

^‡^ OR of children being hospitalized did not change significantly over time (p = 0.62).

* Pearson chi2(8) = 26.69, p = 0.001.

^†^ OR of death was decreased by 8% per one year change in calendar year (OR: 0.92, 95% CI 0.88–0.96, p<0.001).

^β^ No significant temporal trends in length of stay were found (p = 0.46).

^α^ Mechanical ventilation identified with the following ICD-9 procedure codes: 96.71 and 96.72.

^π^ Hemodialysis identified with the following ICD-9 procedure codes: 39.95 and 38.95.

^Ω^ Quan modification of the Deyo-Charlson Comorbidity Index [12,13].

**Fig 1:**
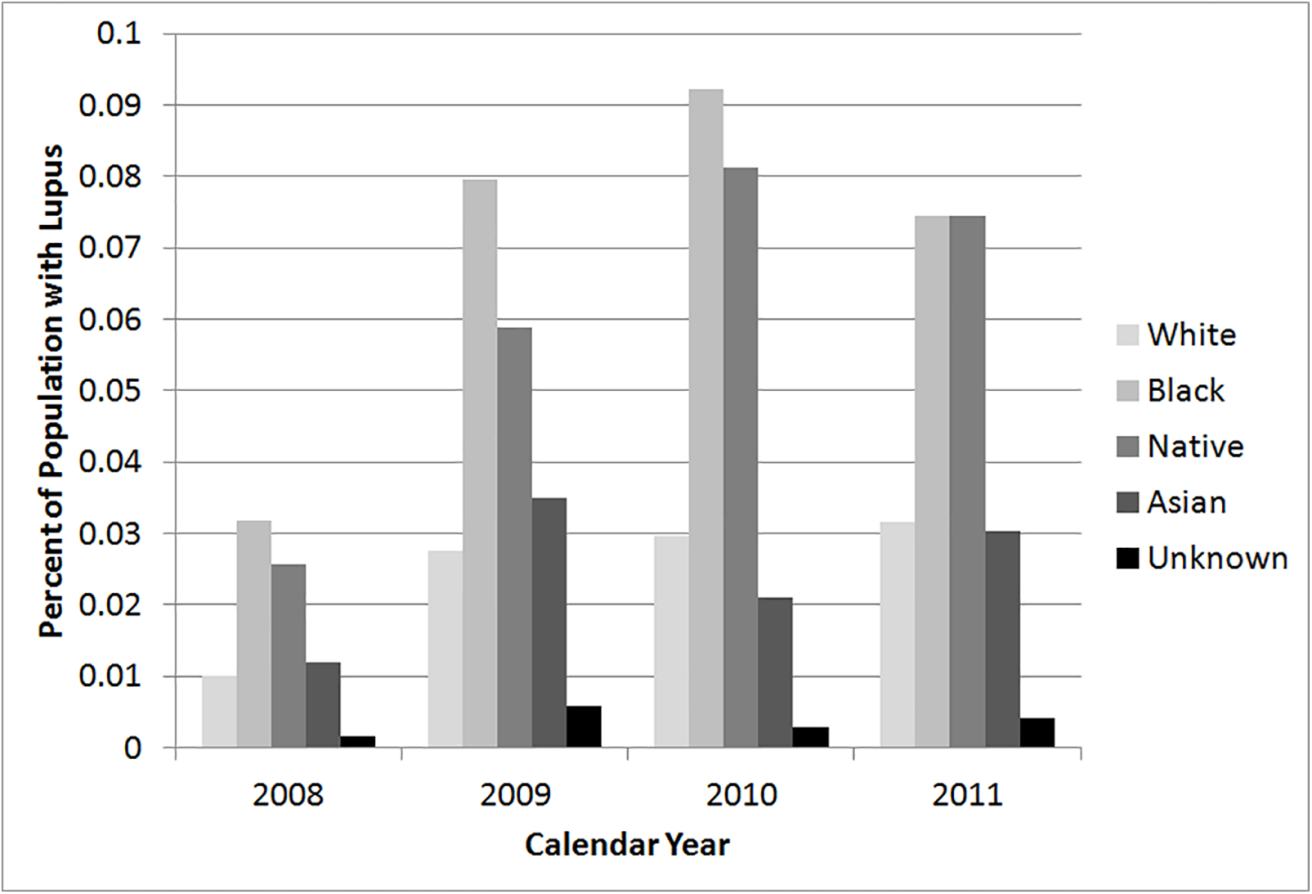
Prevalence of lupus hospitalizations by race based on Washington State population estimates, 2008 through 2011.

**Fig 2:**
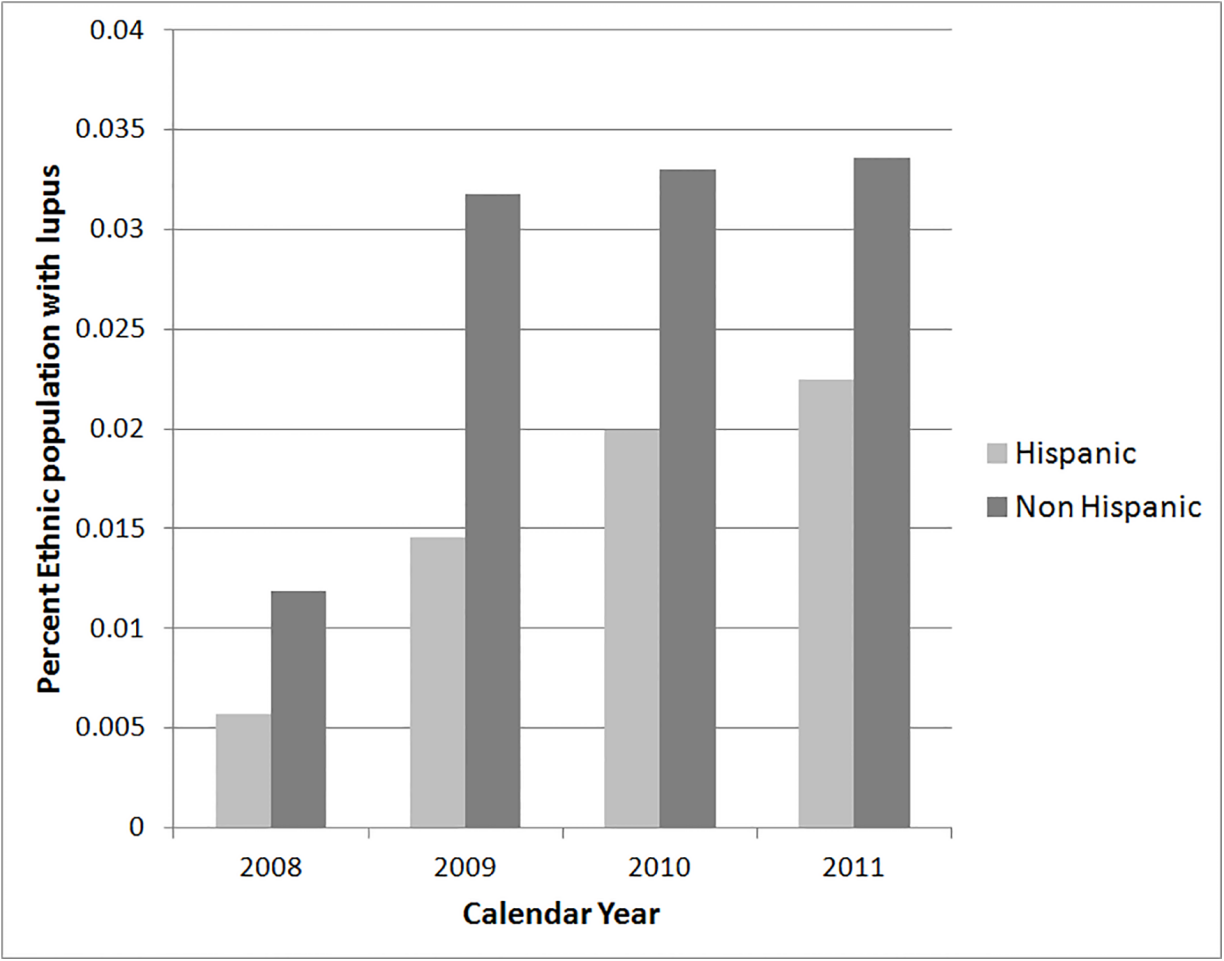
Prevalence of lupus hospitalizations in Hispanics and non-Hispanics based on Washington State population estimates, 2008 through 2011.

We could only evaluate race and ethnicity from 2008–2011. As seen in Fig 1, blacks had the highest prevalence of lupus based on the racial distribution of the total population, followed, in decreasing order, by Native Americans, whites, Asians, and the unknown category. In 2011 the percentage of whites in the total population of Washington was 82.0%, significantly higher than the 63.3% of white hospitalized lupus patients (p<0.001). There was a significantly higher proportion of black lupus patients compared to the proportion of blacks in the Washington census. In 2011 the percentage of blacks in the total population of Washington State was 3.8% compared to the percentage of black lupus patients which was 7.8% (p<0.001). Hispanics had a lower prevalence of lupus hospitalizations than non-Hispanics. In 2011 the percentage of Hispanics in the total population of Washington state was 11.6% compared to the percentage of hospitalized Hispanic lupus patients at 7.2% (p<0.001)(Fig 2).

### Clinical Outcomes

The overall length of stay remained the same over the eight year study period (Table 1). Overall, the median length of stay (LOS) was three days with an interquartile range of two to six days. Over the study period, LOS did differ by year (p = 0.01). This was due primarily to one year—2010. Although LOS did differ by year, we found no significant trend in length of stay (test of trend p = 0.46) over the study period. In a comparison between length of hospitalization stay between females and males, females had a significantly shorter length of stay than males (p = 0.01). The average length of stay for males was 5.2 days while the length of stay for females was on average only 4.9 days. The average length of stay for adults with lupus was longer than children (5.0 days for adults vs 3.5 days for children; median length of stay for adults was 3.0 days compared to 1.0 day for children, p<0.001). We saw no change in the proportion of children compared to adults hospitalized with lupus during the study period (Table 1).

Despite no temporal change in hospital length of stay for patients admitted with a diagnosis of lupus, hospital mortality decreased by 50% during the study period, although the absolute change was small (Table 1). For example, in 2003 the overall hospital mortality for hospitalized lupus patients was 3.1% compared to 1.3% in 2011. We found a significant temporal association for this decreasing event rate over time; the odds of dying during a hospitalization decreased 8% per year during the study period [odds ratio(OR): 0.92 per year, 95% CI 0.88–0.96, p<0.001]. Overall hospital mortality during the entire period was 1.9%. After adjusting for race, the OR did not change (OR of 0.92 per year, 95% CI: 0.88–0.96) (S1 File ).To further assess the role of gender in severity of illness in lupus, we examined whether there were differential hospital mortality rates between males and females. During the study period, more males than females died during their hospitalization with 2.6% of males with lupus dying while only 1.8% of females died during their hospitalization (p = 0.016). Even though males represent only 11.5% of the total number of patients hospitalized with lupus, they accounted for 15.4% of all the deaths. We found a significant association with calendar year and decreasing hospital mortality in both females (OR: 0.92, 95% CI: 0.88–0.96, p<0.001, for each increase in calendar year) and males (OR: 0.89, 95% CI: 0.80–0.99, p = 0.03, for each increase in calendar year). A table comparing those patients who died and those who survived their hospitalization is available in Table A in S1 File. We found no significant difference in mean age from patients hospitalized in 2003 and 2011 (mean difference 0.01 years, 95% CI: + 0.1 to -0.1, p = 0.94).

Because of the concern of the lack of specificity of ICD-9 for identifying cases of lupus[15], we analyzed only those cases in which lupus was the primary diagnostic code. In this restricted analysis, the effects we saw were more pronounced. For each year increase in calendar year, the OR was 0.76 (95% CI 0.62 to 0.92) suggesting a 24% reduction in the odds of death during the hospitalization per year (Tables B and C in S1 File). The subset with lupus as the primary diagnostic code was only 1,634 hospitalizations, or 8.6% of the total of 18,905.

One of the potential concerns with these results is that they could reflect a decreasing severity of illness of the population with lupus. To address this concern, we evaluated the rate of hemodialysis and mechanical ventilation in this population over time. Rates of hemodialysis and mechanical ventilation appeared relatively stable during the time period (Table 1). Adjusting for these specific markers of severity of illness did not change the overall trends we saw in decreasing hospital mortality (adjusting for hemodialysis: OR 0.92, 95% CI: 0.88–0.96 and adjusting for mechanical ventilation: OR 0.91, 95% CI: 0.87–0.95)(see Models 1–3 in Table C in S1 File). Additionally, we assessed the Quan modification of the Deyo-Charlson Comorbidity Index during the study period—this index actually appeared to increase during the time period (Table 1). Adjusting for the index did not significantly impact our results—OR of death per increase in calendar year was 0.90 (95% CI 0.87–0.94) (see Model 4 in Table C in S1 File).

The population of Washington State did grow during the study period from roughly 6.1 to 6.8 million people and the overall rate of lupus hospitalizations also increased from 29.9 to 36.7 hospitalization per 100,000 (p<0.001) (Table D in S1 File). However, when we evaluated a time period with almost no change in the lupus hospitalization rates from 2003–2008 and still noted a reduction in the odds of death during hospitalization for lupus patients (OR = 0.88, 95% CI: 0.82 to 0.95).

## Discussion

In our population based study using ICD-9-CM codes to define hospitalized patients with lupus, we found that despite no significant change in hospital length of stay, hospital mortality has significantly decreased from 2003 to 2011 in Washington State. Absolute hospital mortality remained low during the entire time period. Males had higher hospital mortality during this time period than females, although both populations had decreasing hospital mortality. Hospitalized females outnumber males by a ratio of 9:1. Males, while they less commonly have lupus, have more severe manifestations of the disease, marked by longer hospital stays and higher rates of death during hospitalization.

Lupus remains a challenging disease to treat; the etiology of this disease is still elusive. Prior studies have shown that hospital morbidity and mortality may differ based on gender, race and ethnicity.[1,6,7] While studies have evaluated and documented recent trends in decreasing hospital mortality in children with lupus,[6,7] no prior work has focused on a specific region employing hospital billing data. Our data suggests that improved survival in both children and adults with lupus in Washington State supports a nationwide trend in improved hospital outcomes. Similar trends have been noted in Spain using ICD-9-CM codes to identify cases; however, only males with lupus were noted to have decreased mortality.[16] In Taiwan, ICD-9-CM codes have been used to identify cases of lupus and clarify morbidity associated with ICD-9-CM diagnosis code for lupus complications—these include risk of heart failure, hypertension, osteoporosis, cataracts, glaucoma, dyslipidemia, seizures, encephalopathy, and malignant changes—compared to non-lupus populations.[17]

The increased severity of disease in males that we noted has been investigated in a number of animal studies.[18,19] The role of sex on disease severity was demonstrated by the acceleration of the disease progression and death in males.[20,21] Due to the much smaller male population, studies have had limited power to address factors that contribute higher disease severity after adjusting for renal involvement, central nervous system involvement, ethnicity and socioeconomic status in males including behavioral and adherence factors.[22]

Our analyses noted that a large proportion of patients with lupus are white, corresponding to the predominantly white population of Washington State. When the prevalence of lupus in different racial groups was compared to their racial populations in all of Washington, black patients had the highest prevalence among different racial groups. This finding has been seen in the literature; 0.7% of black adult females as opposed to 0.2% of white adult females are diagnosed with lupus, representing over a three-fold increased risk.[23] Regional differences in race can influence the distribution of patients with lupus. Importantly, the majority of large-scale genetic studies in lupus have been performed in populations of European descent.[24] We found that non-Hispanics had a higher prevalence of being hospitalized with severe lupus compared to Hispanics. These results differed from prior studies that noted similar disease prevalence in Hispanics compared to non-Hispanic whites.[25] Differential severity of disease, socioeconomic differences, or genetic background of Hispanic populations studied (i.e. Hispanics of Puerto Rican ancestry compared Hispanics of Brazilian ancestry) may explain divergent results.[25] Our data does not address disease prevalence in the overall population of lupus patients who do not require hospitalization; recent work has demonstrated an association of socioeconomic status and exacerbations of lupus with disease severity in lupus.[1,6,26] Thus, comparison may be problematic due to the challenges of basing the genetics of a disease on ethnicity.

We showed that lupus hospital mortality has significantly decreased reflecting similar trends observed in a recent analysis of the national Kids’ Inpatient Database.[7] As in our study, these investigators demonstrated stable hospitalization rates and length of stay of patients with billing codes for lupus. In conjunction with these stable temporal trends, they showed a decrease in hospital mortality; the authors attributed this change in clinical outcome to improvements in treatment.[7] Importantly, newer agents have been shown to lead to similar outcomes with reduced toxicity.[27,28]

Our study should be interpreted in light of its limitations. Our results might merely mirror improvements seen in other rheumatologic diseases during this time frame [29]. This interpretation however, would not influence the message to patients with lupus. Some of the admissions may have been recurrent admissions of the same patient. This raises the concern that hospitalizations were not independent [30]. If patients were hospitalized multiple times within the eight year time period of this analysis, attributes including their race and gender would have been repeated in the database yet counted as individual patients. This should not have affected the trends due to the total number of years and admissions available for this study. It is unlikely that annual readmission rates changed dramatically during this time period due to the relatively stable proportion of lupus hospitalizations (Table A in S1 File). Additionally, inability to account for repeated hospitalizations within the same subject primarily impacts the estimates of the 95% CI’s. The point estimates and the restricted analyses suggest that repeat hospitalizations did not markedly impact our data. Next, the assessment of race and ethnicity was based on self-report and may have changed over time. Also, groups such as Hispanic blacks would be misclassified using our methodology; however, this population is likely to be very small in Washington State. Next, some subgroups of patients are small, and analyses of these groups may have been more strongly influenced by repeat hospitalizations. Also, the differences in how race and ethnicity are categorized in the census and the HCUP data differed; our groupings may not have been completely comparable in the two data sets, introducing error in our estimates. Also we did not analyze associated diagnoses (other than evaluating the use of dialysis and mechanical ventilation) that led to hospital admission (e.g. acute kidney injury, end-stage renal disease); these diagnoses could affect the rates of hospitalization and modulate the analyses of race/ethnicity. We were able to adjust for comorbid illnesses and demonstrated that this did not influence our results. Last, we employed ICD-9-CM codes to identify patients admitted to Washington State hospitals with lupus. This could lead to misclassification of disease. However, prior work has validated the use of ICD-9-CM codes for identifying hospitalized lupus patients.[31] Other work has questioned the positive predictive value of ICD-9-CM coding in administrative data sets to study lupus finding a positive predictive value as low as 50% [32] when using ICD-9-CM coding to identify cases with lupus from the general population. However, the authors noted that algorithms to restrict the ICD-9-CM diagnosis yield more specific but likely less generalizable results. Also our work applies to only those lupus patients requiring hospitalization thus representing a more severe subset of patients. Our work may also not generalize beyond Washington State, however the reduction in pediatric lupus hospital mortality noted nationwide by Knight et al. [7] supports our conclusions. Despite the limitations noted above, the large sample size and the number of years of data available allow for meaningful comparisons in hospitalizations lupus patients in Washington State.

## Conclusion

Using a statewide database designed for epidemiologic research using diagnostic and procedure codes for identification of diagnoses and the Washington data from the US census, we described changes in demographics of patients hospitalized for lupus in Washington State. We also found dramatic reductions in hospital mortality despite no clear trends in hospital length of stay. Further study is required to understand what specific changes in care may have driven this reduction in hospital mortality.

## Supporting Information

S1 Fig Proportion of Hospitalized Patients by Gender: figure notes the gender distribution by age category. Proportion of females noted in green stacked above proportion of males in blue.
(JPG)

S2 Fig Distribution of total number of Patients hospitalized with SLE in Washington by age category. Females are noted in the white bars; males are noted in the superimposed light blue bars.
(JPG)

S1 File Contains additional sensitivity analyses. It also contains Tables A-D. Table A contains a comparison of those lupus hospitalizations that resulted in death compared to those that did not during the entire study period. Table B contains a comparison of those hospitalizations that have lupus as a primary diagnostic code by year compared with those with a secondary diagnostic code. This table notes the relatively stable rate of hospitalization where lupus was the primary diagnostic code. Table C contains adjusted regression models assessing temporal change in the OR of hospital death. Multivariate logistic regression models evaluate simultaneously the impact of calendar year adjusting for markers of comorbid illness (hemodialysis and mechanical ventilation) in addition to employing the Quan modification of the Deyo-Charlson Combidity Index, a validated index to account for comorbid illnesses in hospitalized patients. Table D contains the proportion of lupus hospitalizations, to total Washington State Population. Washington State population was based on census data.
(DOCX)
