# Correction: Significant Reductions in Mortality in Hospitalized Patients with Systemic Lupus Erythematosus in Washington State from 2003 to 2011

**DOI:** 10.1371/journal.pone.0140698

**Published:** 2015-10-09

**Authors:** Louisa B Goss, Justin R Ortiz, Daryl M Okamura, Kristen Hayward, Christopher H Goss

## Notice of Republication

This article was republished on September 30, 2015, due to the release of confidential or copyrighted material. Please download this article again to view the correct version.
