# Supplementary figures and images for: Significant Reductions in Mortality in Hospitalized Patients with Systemic Lupus Erythematosus in Washington State from 2003 to 2011
Source: PLoS One. 2015 Jun 18;10(6):e0128920. doi: 10.1371/journal.pone.0128920 (PMC4473009; doi:10.1371/journal.pone.0128920)

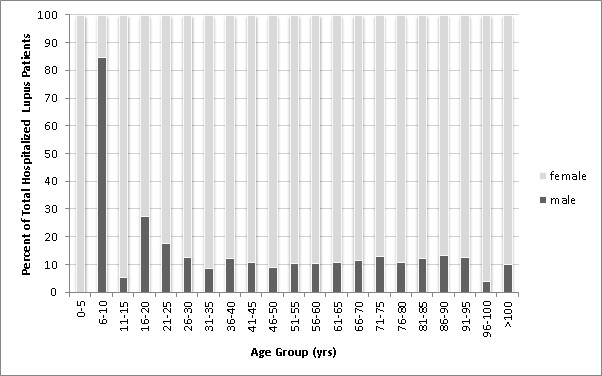

Supplement: S1 Fig — Proportion of females noted in green stacked above proportion of males in blue. (JPG) [file pone.0128920.s001.jpg]

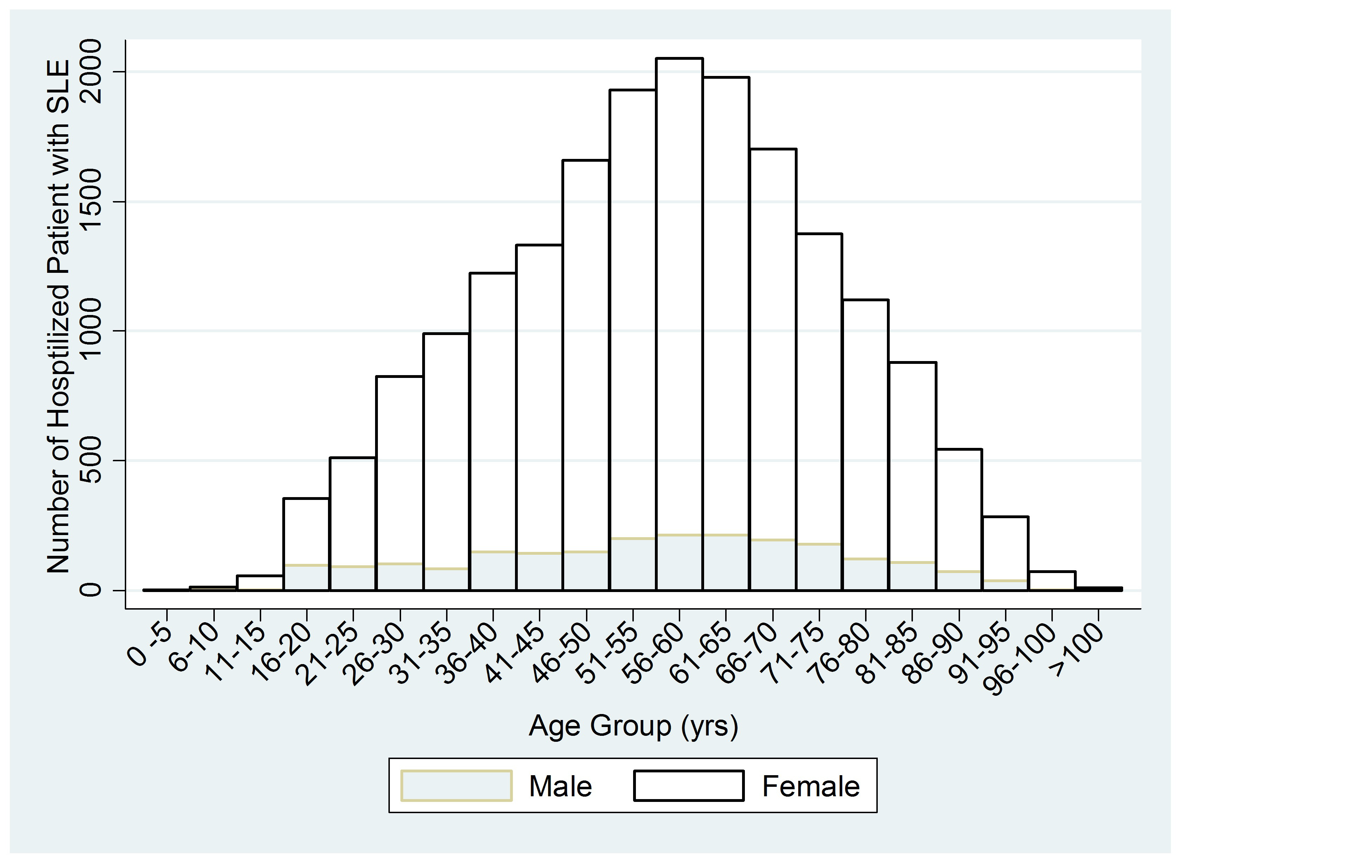

Supplement: S2 Fig — Females are noted in the white bars; males are noted in the superimposed light blue bars. (JPG) [file pone.0128920.s002.jpg]
