# Supplementary material for: Significant Reductions in Mortality in Hospitalized Patients with Systemic Lupus Erythematosus in Washington State from 2003 to 2011
Source: PLoS One. 2015 Jun 18;10(6):e0128920. doi: 10.1371/journal.pone.0128920 (PMC4473009; doi:10.1371/journal.pone.0128920)
Supplement: S1 File — It also contains Tables A-D. Table A contains a comparison of those lupus hospitalizations that resulted in death compared to those that did not during the entire study period. Table B contains a comparison of those hospitalizations that have lupus as a primary diagnostic code by year compared with those with a secondary diagnostic code. This table notes the relatively stable rate of hospitalization where lupus was the primary diagnostic code. Table C contains adjusted regression models assessing temporal change in the OR of hospital death. Multivariate logistic regression models evaluate simultaneously the impact of calendar year adjusting for markers of comorbid illness (hemodialysis and mechanical ventilation) in addition to employing the Quan modification of the Deyo-Charlson Combidity Index, a validated index to account for comorbid illnesses in hospitalized patients. Table D contains the proportion of lupus hospitalizations, to total Washington State Population. Washington State population was based on census data. (DOCX) [file pone.0128920.s003.docx]

Supporting Information

Title: Dramatic reductions in mortality in hospitalized patients with systemic lupus erythematosus in Washington State from 2003 to 2011

Author List in Order:

Louisa B Goss^1^; Justin R Ortiz^1,2^ ; Daryl M Okamura^3,4^ ; Kristen Hayward^4,5^ ; Christopher H Goss ^1,5,6^

Institutions:

1. Department of Medicine, University of Washington, Seattle WA USA
2. Department of Global Health, University of Washington, Seattle WA USA
3. Department of Pediatrics, Division of Nephrology, University of Washington, Seattle WA USA
4. Seattle Children’s Research Institute, Seattle WA USA
5. Department of Pediatrics, Division of Rheumatology, University of Washington, Seattle WA USA
6. Department of Pediatrics, Division of Pulmonary, University of Washington, Seattle WA USA

Results

To assess whether changes in racial groups impacted our findings we first adjusted for the largest racial minority in the data set, those that are black. This population has been noted in the literature to be associated with worse clinical outcomes. When adjusting for the impact of being categorized as black compared to all other racial groups, we found that the odds ratio (OR) for death during the hospitalization for each additional calendar was an OR of 0.92 per year (95% CI: 0.88-0.96). In the adjusted model, being black was associated with a non-statistically significant increase in OR for death (OR 1.01. 95% CI: 0.55 to 1.87). This effect may have been due partly to the low percentage of the population which self-reported as black.

To address the potential for ICD-9 diagnostic codes capturing disease unrelated to the primary disease of interest, we looked at only those hospitalizations in which lupus was the primary diagnostic code. Here, we found an even stronger association of hospital mortality and calendar year. As noted in Table B, the proportion of hospitalizations categorized as primary diagnosis of lupus represented only 8-10% of the hospitalizations. Despite this limited subset, the change in hospital mortality per calendar year was associated with a greater decreased OR of death (0.76, 95% CI: 0.62 to 0.92).

Table C addresses a number of sensitivity analyses and multivariable adjusted analyses to assess the impact of disease severity and comorbidities on the temporal improvement in hospital mortality we have demonstrated. As noted in Table C, neither the use of dialysis or mechanical ventilation accounted for the improvement in hospital mortality. Both procedures were associated with an increased hospital mortality, with mechanical ventilation (both short-term and long-term) being associated with the highest OR of death during the hospitalization. Last, we employed the Quan adaptation of the Deyo-Charlson Index to adjust for comorbid illnesses (REF: Quan H et al. Med Care 2005;43: 1130–1139; Quan H,et al. Am J Epidemiol. 2011 Mar 15;173(6):676-82). This index performs better than the well validated Charlson Comorbidity Index (CCI) and the Deyo-Charlson Comorbidity Index.

Table A in S1 File: Comparison of those lupus hospitalizations that resulted in death compared to those that did not during the entire study period.

| **Clinical Characteristic** | **Lupus Hospitalizations in which the patient died** | **Lupus Hospitalizations in which the patient survived** |
| --- | --- | --- |
| N | 363 | 18,542 |
| Female Gender (%) | 307 (1.8) | 16,433 (98.2) |
| Male Gender (%) | 56 (2.6) | 2,109 (97.4) |
| Mean Age (SD) | 66.56 (16.64) | 51.00 (17.95) |
| Children ^¶^ (%) | ≤ 10 (≤ 1.6)* | 613 (99.4) |
| Adult (%) | 359 (2.0) | 98.0) |
| Ethnicity |  |  |
| Hispanic (%) | ≤ 10 (≤ 2.1)* | 456 (98.1) |
| Race |  |  |
| Caucasion (%) | 280 (2.0) | 13,589 (98.0) |
| Black (%) | 11 (1.5) | 702 (98.5) |
| Mechanical Ventilation (%) | 167 (29.6) | 397 (70.4) |
| Hemodialysis (%) | 33 (2.8) | 1,144 (97.2) |
| Mean CCI ^‡^ (SD) | 3.5 (2.2) | 2.3 (1.6) |

¶ Less than 18 years of age

‡ Quan modification of the Deyo-Charlson Comorbidity Index (CCI) (REF: Quan H et al. Med Care 2005;43: 1130–1139; Quan H,et al. Am J Epidemiol. 2011 Mar 15;173(6):676-82

* Per the provisions of the SID Data Use Agreement (DUA), no data observation involving less than or equal to 10 observations are to be published.

Table B in S1 File: Comparison of those hospitalizations that have lupus as a primary diagnostic code by year compared with those with a secondary diagnostic code. This table notes the relatively stable rate of hospitalization where lupus was the primary diagnostic code.

| Year | Hospitalizations of patients with lupus as a primary diagnostic code (% total) | Hospitalizations of patients with lupus as a secondary diagnostic code (% total) |
| --- | --- | --- |
| 2003 | 151 (8.3) | 1,678 (91.7) |
| 2004 | 147 (8.4) | 1,596 (91.6) |
| 2005 | 166 (8.8) | 1,727 (91.2) |
| 2006 | 206 (10.1) | 1,829 (89.9) |
| 2007 | 175 (8.6) | 1,859 (91.4) |
| 2008 | 184 (8.7) | 1,923 (91.3) |
| 2009 | 197 (8.5) | 2,113 (91.5) |
| 2010 | 198 (8.1) | 2,225 (91.9) |
| 2011 | 210 (8.4) | 2,294 91.6) |

Table C in S1 File: Adjusted regression models assessing temporal change in the OR of hospital death. Multivariate logistic regression models evaluate simultaneously the impact of calendar year adjusting for markers of comorbid illness (hemodialysis and mechanical ventilation) in addition to employing the Quan modification of the Deyo-Charlson Combidity Index, a validated index to account for comorbid illnesses in hospitalized patients.

| Model | Covariates | Adjusted Odds Ratio (OR) ^‡^ | 95 % CI |
| --- | --- | --- | --- |
| Model 1 | Calendar year ^¶^ | 0.92 | 0.88 to 0.96 |
|  | Hemodialysis (HD)  Reference: no HD | ---- | ---- |
|  | HD+ | 1.51 | 1.04 to 2.16 |
| Model 2 | Calendar year ^¶^ | 0.91 | 0.87 to 0.95 |
|  | Mechanical Ventilation (MV)  Reference: no MV | ---- | ---- |
|  | MV+ | 39.6 | 31.4 to 49.9 |
| Model 3 | Calendar year ^¶^ | 0.91 | 0.87 to 0.95 |
|  | Hemodialysis (HD)  Reference: no HD | ---- | ---- |
|  | HD | 1.10 | 0.74 to 1.64 |
|  | Mechanical Ventilation (MV)  Reference: no MV | ---- | ---- |
|  | MV+ | 39.4 | 31.3 to 49.7 |
| Model 4 | Calendar year ^¶^ | 0.92 | 0.88 to 0.96 |
|  | Charlson Comorbidity Index * | 1.34 | 1.28 to 1.39 |

¶ OR of death associated with hospitalization with each one year increase in calendar year

‡ OR of death associated with hospitalization

* Quan modification of the Deyo-Charlson Comorbidity Index (REF: Quan H et al. Med Care 2005;43: 1130–1139; Quan H,et al. Am J Epidemiol. 2011 Mar 15;173(6):676-82

Table D in S1 File: Proportion of lupus hospitalizations, to total Washington State Population. Washington State population was based on census data.

| Year | Hospitalizations of patients with lupus | Washington State Population | Cases of lupus per 100,000 population |
| --- | --- | --- | --- |
| 2003 | 1829 | 6,113,262 | 29.9 |
| 2004 | 1743 | 6,184,289 | 28.2 |
| 2005 | 1893 | 6,261,282 | 30.2 |
| 2006 | 2035 | 6,372,243 | 31.9 |
| 2007 | 2034 | 6,464,979 | 31.5 |
| 2008 | 2107 | 6,566,073 | 32.1 |
| 2009 | 2310 | 6,664,195 | 34.7 |
| 2010 | 2450 | 6,743,636 | 36.3 |
| 2011 | 2504 | 6,823,267 | 36.7 |
